# Advancing the application of systems thinking in health: analysing the contextual and social network factors influencing the use of sustainability indicators in a health system – a comparative study in Nepal and Somaliland

**DOI:** 10.1186/1478-4505-12-46

**Published:** 2014-08-26

**Authors:** Karl Blanchet, Jennifer Palmer, Raju Palanchowke, Dorothy Boggs, Ali Jama, Susan Girois

**Affiliations:** International Centre for Evidence in Disability, London School of Hygiene and Tropical Medicine, Keppel St, Bloomsbury, London WC1E 7HT UK; Handicap International, Narayan Gopal Chowk Sallaghari, PO Box 10179, Kathmandu, Nepal; Handicap International, 9 Rushworth Street, London, SE1 UK; Disability Action Network, Hargeisa, Somaliland; Norfolk Community Services Board, 6401 Tidewater Dr, Norfolk, VA 23509 USA

**Keywords:** Disability, Health systems, Nepal, Physical rehabilitation, Social network analysis, Somaliland, Sustainability, Systems thinking

## Abstract

**Background:**

Health systems strengthening is becoming a key component of development agendas for low-income countries worldwide. Systems thinking emphasizes the role of diverse stakeholders in designing solutions to system problems, including sustainability. The objective of this paper is to compare the definition and use of sustainability indicators developed through the Sustainability Analysis Process in two rehabilitation sectors, one in Nepal and one in Somaliland, and analyse the contextual factors (including the characteristics of system stakeholder networks) influencing the use of sustainability data.

**Methods:**

Using the Sustainability Analysis Process, participants collectively clarified the boundaries of their respective systems, defined sustainability, and identified sustainability indicators. Baseline indicator data was gathered, where possible, and then researched again 2 years later. As part of the exercise, system stakeholder networks were mapped at baseline and at the 2-year follow-up. We compared stakeholder networks and interrelationships with baseline and 2-year progress toward self-defined sustainability goals. Using in-depth interviews and observations, additional contextual factors affecting the use of sustainability data were identified.

**Results:**

Differences in the selection of sustainability indicators selected by local stakeholders from Nepal and Somaliland reflected differences in the governance and structure of the present rehabilitation system. At 2 years, differences in the structure of social networks were more marked. In Nepal, the system stakeholder network had become more dense and decentralized. Financial support by an international organization facilitated advancement toward self-identified sustainability goals. In Somaliland, the small, centralised stakeholder network suffered a critical rupture between the system’s two main information brokers due to competing priorities and withdrawal of international support to one of these. Progress toward self-defined sustainability was nil.

**Conclusions:**

The structure of the rehabilitation system stakeholder network characteristics in Nepal and Somaliland evolved over time and helped understand the changing nature of relationships between actors and their capacity to work as a system rather than a sum of actors. Creating consensus on a common vision of sustainability requires additional system-level interventions such as identification of and support to stakeholders who promote systems thinking above individual interests.

## Introduction

Health systems strengthening is becoming a key component of development agendas for low-income countries worldwide. As a means to achieve this, systems thinking provides perspectives on how health systems can be assessed [1], recognizing non-linearity, complexity, heterogeneity, uncertainty, and ambiguity of real-world settings [1–4]. The 2009 Flagship Report from the Alliance for Health Policy and Systems Research proposes “Ten Steps to Systems Thinking”, emphasizing the roles of diverse stakeholders in designing solutions to system problems, including sustainability [1]. Studying information flow mechanisms between actors and within networks can help us to understand decision-making processes, as well as the social processes which influence the resilience of socio-ecological systems (including health systems). Asch [5] showed that individuals’ decisions in an unpredictable world are often based on peers’ opinions and actions. Interactions and collaboration between stakeholders depend on various social factors, such as trust, conflict resolution, and knowledge integration [6], and also on circulation of information within social networks [7, 8].

The structure of social networks influence individual actors’ capacity to respond to the needs of the system as a whole [9, 10]. It follows that understanding system stakeholder networks may be important when analysing how information on system sustainability can be used by the actors of the system to make informed decisions [11, 12]. However, the structure of social networks may only be one amongst other factors contributing to the use of data in decisions. Understanding the dynamics of systems therefore requires combining several methodologies to capture the complexity of health programmes, the embeddedness of systems within other systems, and the multi-layered governance of health systems [13–15].

In this paper, we build on previous work to introduce systems thinking among local stakeholders of the physical rehabilitation system in Nepal and Somaliland [16]. Although sustainability has been at the heart of recent international health programmes and policies, the meaning of sustainability remains unclear and confusing to most public health professionals [17, 18]. The current challenges for policy-makers and researchers are to translate the concept of sustainability into concrete indicators [19], which will help policy makers and health service managers make public health and management decisions [20]. However, in order to be successful, such a process should also attend to the political tensions involved in “knowledge production” and “norm creation” inherent to sustainability planning in any system. We used the Sustainability Analysis Process (SAP), a system-oriented tool, which encourages participants to arrive at consensus about system boundaries, define sustainability, and identify measurable indicators for a sustainable system [21].

During this process, the concept of sustainability is upheld as normative [22]. The process also avoids decisions taken by a limited number of “experts”. This implies that those participating in the consensus building process are not only acting in their technical expert capacity, but also as “political actors” taking normative decisions on what aspects to uphold [23–26]. Involving a wide range of diverse actors of the health system, including users, of course raises practical problems. For example, the imbalance of power existing between different groups of stakeholders [27, 28] means that some topics can be neglected during this process because people who defend them do not receive enough consideration within the group [29–31]. The final “step” of the process additionally includes piloting and re-visiting the measurement of sustainability indicators to judge their fit outside of a workshop setting. The consensus building process needs to capture the tensions between “knowledge production” and “norm creation” in a particular context.

Along with convening and observing sustainability analysis workshops in each setting, we sought to analyse the contextual factors and the characteristics of the social networks to identify the influences affecting actors’ decisions about using sustainability data or not [32, 33].

The objective of this paper is to compare the definition and use of sustainability indicators developed through the SAP in two rehabilitation sectors, one in Nepal and one in Somaliland, and analyse the contextual factors (including the characteristics of system stakeholder networks) influencing the use of sustainability data.

## Methodology

In order to capture social phenomena such as management decisions and interactions between individuals, an in-depth qualitative research approach was adopted. According to Fitzpatrick and Boulton ([34] p. 107), qualitative research “*is used where it is important to understand the meaning and interpretation of human social arrangements such as hospitals, clinics, forms of management or decision making*”. In real-life contexts, multiple case study designs are known to be appropriate for understanding and interpreting complex causal links in natural setting interventions [35, 36]. We combined three different methods; we used stakeholder network analysis, and the SAP at baseline (2010) and at a 2-year follow-up (2012). Interviews with key informants lent depth to the observations, the analysis and helped understand the relationship between the structure of the network, the contextual factors, and the use (or not) of sustainability indicators. Each of these three methods is described below.

### Stakeholder network analysis

Stakeholder network analysis was used to map key stakeholders in the physical rehabilitation system and identify network characteristics. The analysis was conducted in both Nepal and Somaliland in 2010 and again in 2012, and is detailed elsewhere [37, 38]. In summary, stakeholder network analysis consists of three stages: (i) describing the set of stakeholders in the network/system (using interviews with stakeholders and document review), (ii) characterising the relationships between stakeholders (interviews), and (iii) analysing the structure of the network/system (using software, see below) [38]. Stakeholders were defined as persons, informal groups of people, or formal organisations who may influence the sustainability of the system through their interactions and individual or collective actions [39–41]. Relationships between actors can be of different kinds and depend on various social factors such as trust, conflict, or knowledge sharing [42]. However, all these social factors are interdependent with one key process: the circulation of information between and within social networks [43, 44]. The second stage of stakeholder network analysis consisted of identifying the existence of flows of information between actors or, in other words, the demand (receiving information) and supply (providing information) of information between individuals. This information was collected through interviews. Data collected through interviews were recorded in an information flow matrix:one matrix on the demand for information and a second one on the supply of information. Each respondent thus generated a row of “ones” and “zeros” for each of the two network relations (demand and supply of information): “one” symbolising the existence of demand/supply of information and “zero” signifying no information flow between the two actors. The final matrix was then analysed with the software UCINET to generate statistics about the network structure (Table 1) [39, 45], to visually represent relationships within the network and to identify network brokers, who control the flow of information and/or resources within the network [46].

**Table 1:** **Definitions of key network characteristics measured**

Characteristic	Definition
Betweenness	Indicator of centrality of the network as a whole [39]. Corresponds to the number of direct ties a stakeholder has with any other actor compared to the total number of direct ties [39]. The higher the percentage of betweenness, the more centralised the network.
Density	Indicator of network cohesiveness [39]. Defined as the number of existing ties divided by the number of possible ties between stakeholders. Reported on a scale ranging from 0 (no ties at all) to 1 (all actors are connected to all others).

### Sustainability analysis process (SAP)

The SAP is a participatory method based on systems thinking, which combines the Process Analysis Method five-step approach [47, 48] with a conceptual framework, the Sustainability Framework, which was applied and tested in international health [49, 50]. The five components of the Sustainability Framework, which were used in our study, are: health outcomes, service delivery, organisational capacity and viability, community capacity and context [50]. The SAP also involves five steps, as follows: i) Establish a common understanding of the rehabilitation system in the local context; ii) Define system boundaries; iii) Develop a common vision of sustainability; iv) Select measurable sustainability indicators for the local system; v) Collect baseline indicator data [21, 51].

In both Nepal and Somaliland, the SAP method was implemented during a three-day workshop sponsored by Handicap International in 2010 with key stakeholders involved in the physical rehabilitation system. Participants were purposively selected by Handicap International and Naspir, the national federation of rehabilitation providers in Nepal, and by the two national rehabilitation providers in Somaliland. The first list of participants was shared with and reviewed by the two investigators (KB and JP) in relation with the diversity within the rehabilitation sector. The participants included representatives from the Ministry of Health and/or Ministry of Social Affairs, regional health authorities, selected rehabilitation professional staff (physical therapists and orthopaedic technicians), rehabilitation centre managers, representatives of disabled people’s organizations, and representatives of international donors and non-governmental organizations (NGOs) involved locally.

Two-year follow-up workshops were held in both countries in 2012. All the organisations that were represented at the first workshop were represented at the follow-up workshop. After the SAP was reviewed, workshop participants discussed key events that influenced the sustainability of the rehabilitation sector during the intervening 2 years. Sustainability indicators were re-measured, where possible, and participants reflected on progress toward self-defined sustainability goals.

### In-depth interviews and observations

Analytic narrative was used to provide explanations of unique events and outcomes, and can serve the interests of the social researchers who try to describe what events take place, why, as well as their significance to actors within a system [52]. Analytic narrative is considered “*a useful tool for assessing causality in situations where temporal sequencing, particular events, and path dependence must be taken into account*” ([53] p. 1,164). The analytic narrative approach consists of interviewing key actors and understanding their goals, and the main factors influencing their behaviour and decisions [52]. It also requires analysis of the interactions between actors and their impact on institutional settings: “*The emphasis is on identifying the reasons for the shift from an institutional equilibrium at one point in time to a different institutional equilibrium at a different point in time*” ([54], p. 11).

Information was collected from key informants on experiences with collecting and analysing sustainability information by individual actors/organisations. Interviews were conducted in private and participants were assured of confidentiality to encourage participants to share potentially sensitive issues and insights. Interviews were recorded. Transcripts and notes from each interview and group discussion were read in their entirety before coding line-by-line to identify and label ideas and meanings conveyed in each small section of text. These codes were then grouped and labelled to reflect broader themes within the data. Further additions and revisions to the coding framework were made on a continual basis as higher level constructs were generated, through reviewing emerging themes and interpreting them in relation to stakeholder network analysis findings.

## Results

### The 2010 (baseline) physical rehabilitation stakeholder networks in Nepal and Somaliland

The structure and properties of the physical rehabilitation stakeholder networks in Nepal and Somaliland are described and compared elsewhere [16]. Key notions are summarised as follows.

In 2010, the social network of rehabilitation actors in Nepal (56 actors) was over twice as large as the network in Somaliland (22 actors) and there were substantial differences in the types of actors involved in service delivery and system governance. Notably, in Nepal, three ministries – the Ministry of Health and Population, the Ministry of Women, Children and Social Welfare, and the Ministry of Peace and Reconstruction – were directly involved in governance of physical rehabilitation services. In Somaliland, the Ministry of Public Health was solely in charge of rehabilitation services, although the Ministry of Labour and Social Affairs coordinated broader disability issues.

Country differences in the involvement of local NGOs and disabled people’s organisations were also marked. In Nepal, local organisations provided services and directly managed rehabilitation centres. These organisations included professional associations (Nepal Physical Therapy Association and Prosthetist and Orthotist Society of Nepal) and disabled people’s organisations. In Somaliland, a much narrower range of actors delivered rehabilitation services. Specifically, two non-profit rehabilitation organisations (Disability Action Network (DAN), supported by Handicap International and the Somaliland Red Crescent Society (SRCS), supported by the International Red Cross Movement) were responsible for the entirety of rehabilitation service delivery. Both organisations in Somaliland were based in the capital city with complementary networks of partners and providers in the periphery. Disabled people’s organisations were not involved in service delivery, nor did they play a role in advocating for rehabilitation resources.

**Figure 1:**
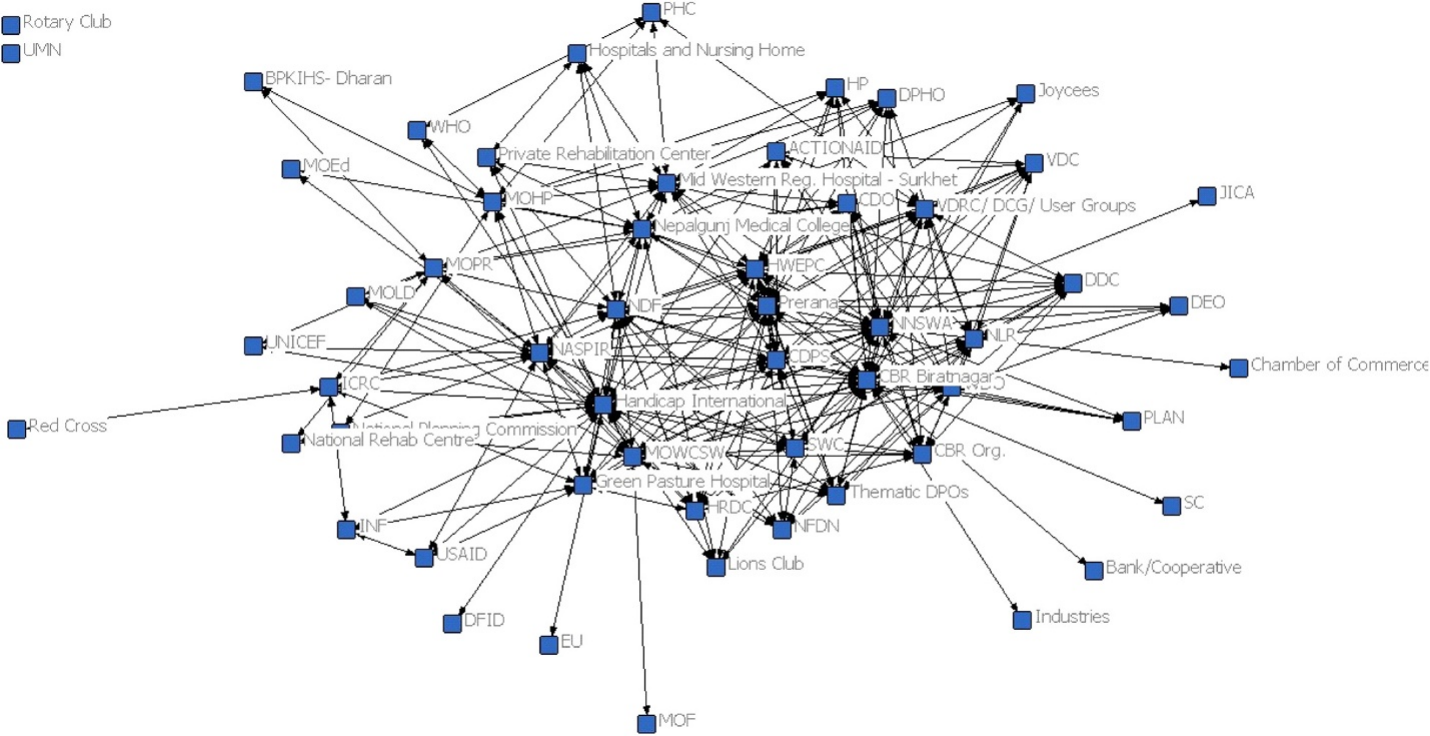
**The physical rehabilitation stakeholder network of Nepal in 2010 (baseline).**

**Figure 2:**
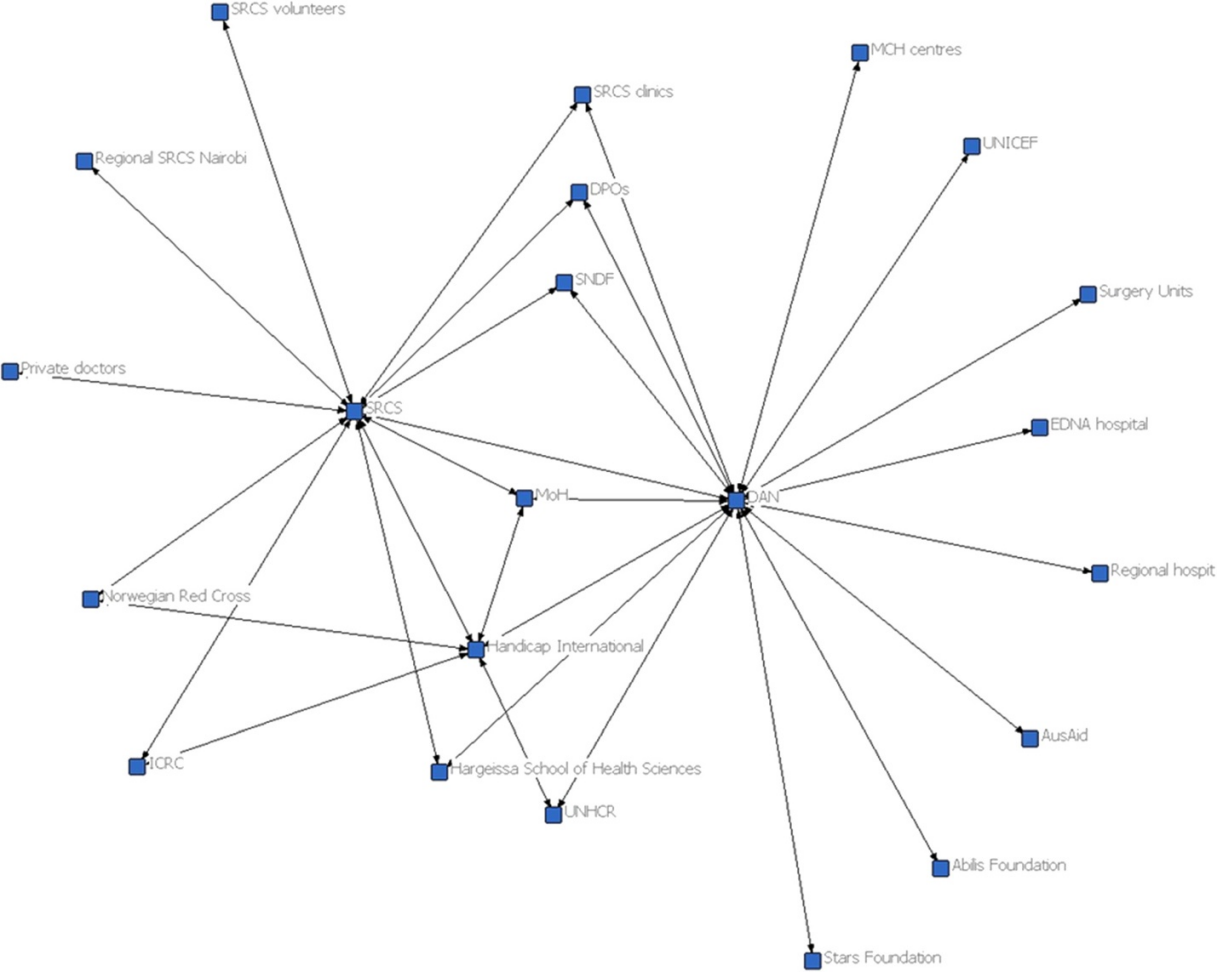
**The physical rehabilitation stakeholder network in Somaliland in 2010 (baseline).**

In 2010, Somaliland’s system stakeholder network was four times more centralised than in Nepal. Nepal’s network density was twice as great as in Somaliland (0.2 in Nepal compared to 0.1 in Somaliland) (Figures 1 and 2). The density of a network is the proportion of all possible ties between actors that are actually present. A centralised stakeholder network, such as in Somaliland, is thought to facilitate communication and innovation, as only a limited number of key actors are involved [39, 55]. On the other hand, centralized networks can easily generate bottlenecks if any of the key stakeholders (brokers) block diffusion of information and/or resources. In a dense network, such as in Nepal, the circulation of information between actors is also rapid but with a much lower risk of bottlenecks.

### The 2010 (baseline) self-defined sustainability indicators in Nepal and Somaliland

Discussions that took place during the 2010 SAP workshops differed between the two countries. A comparison of 10 key self-defined sustainability indicators in each of the two countries is provided in Table 2 (the full list of indicators chosen is available in [56, 57]). In Somaliland, discussions about indicators, their measurement, and recommendations for the system focused on the two rehabilitation facilities existing in that country. Being at the centre of the network, the sustainability of these organisations highly affected the sustainability of the overall system. Sources of instability in the system consisted of the lack of long-term financial resources for centres in the capital as well as poor access to service users outside the capital. In Nepal, a number of rehabilitation centres existed, but populations living in remote areas were not reached with the level of resources and investment at that time. Sustainability indicator discussions in Nepal therefore focused largely on mainstreaming physical rehabilitation into priority health and social programs, while also transferring some services to other actors at the community level.

**Table 2:** **Example of 10 key self-defined sustainability indicators for the physical rehabilitation system in Somaliland and Nepal, by sustainability component**

Sustainability components	Sustainability indicators	
	Somaliland	Nepal
**Rehabilitation outcomes or outputs**	Percent of people with disabilities (PWDs) entering the centre and whose needs were fulfilled	Number of treatment sessions delivered per month
	Percent of PWDs who were referred to other services	Number of prostheses and/or theses produced every year
**Service provision**	Number of physiotherapists and prosthetics and orthotics technicians (P&O) in Somaliland	Percent of centres of who have at least 1 category I P&O
	Percent of regions with at least 2 PT Assistants and 2 P&O Assistants	Number of CAT I P&O who need to be trained by 2015
**Organisational and financial capacity**	Number of different sources of funding	Percentage of catchment districts referring patients to centres
	Percent of staff with job descriptions	Number of female community health volunteers trained in identification of disabilities
**Community capacity**	Percent of PWDs and carers who know the existence of rehabilitation centres	Percent of districts with District Disability Rehabilitation Committees and Village Disability Rehabilitation Committee
	Percent of assessment and planning exercises involving service users	Percent of Disabled People Organisations that have action plans
**Enabling environment**	United Nations Level of Security	Percent of funding allocated by Government to rehabilitation
	Percent of costs covered by Government	Existence of a national action plan on rehabilitation

In terms of physical rehabilitation goals, network members in both Somaliland and Nepal focused on increased access to and coverage of rehabilitation services across regions (e.g., percent of people with disabilities by rehabilitation centre whose physical rehabilitation needs have been met). In Somaliland, the rehabilitation providers were concerned about the centralisation of services in the capital and the provinces being underserved due to insecurity outside Hargeisa, the capital, restricting both expansion of service provision and the ability of patients to complete referrals made from the periphery.

In terms of service provision, both stakeholder groups recognized that limited numbers and poor geographic distribution of rehabilitation professionals would threaten the sustainability of their system. In Somaliland, network members emphasized the need for professionals to provide services outside the capital city by analysing human resources regionally. In Nepal, stakeholders took a more systemic view and identified the total number of rehabilitation professionals who needed to be trained in the country over the next few years as a sustainability indicator.

In terms of organisational and financial capacity, Somaliland network stakeholders placed strong emphasis on the financial autonomy of rehabilitation service providers (e.g., number of different sources of funding) and team management (e.g., percent of staff with job descriptions or number of coordination meeting per year). One of the two service providers in Somaliland was approaching the end of a funding cycle and had no certainty that international support would continue after the end of 2011. Hence, sustainability indicators specified diverse funding sources and reduced financial risk. Some actors identified the emergence of donors in the domestic and diaspora private sector as an attractive means to diversification, particularly as trust in the political will of the Ministry of Health (a potential influential actor within the network) to intervene in rehabilitation was low.

In Nepal, organisational and financial sustainability goals are aimed at integrating physical rehabilitation into national policies and other programmes (e.g., disability integrated into female community health volunteer activities). Mainstreaming physical rehabilitation into other social or health systems represented an opportunity to secure resources that were not available in an isolated rehabilitation system. Furthermore, network members in Nepal recognised the need to associate with actors outside their system working at the community level to increase coverage of services.

In terms of community capacity, the choice of sustainability indicators in both countries reflected the level of cohesiveness between the rehabilitation services and community-based organisations. In Somaliland, network members defined “community” as the users of the rehabilitation services, and community participation was described in terms of the financial capacity of users to pay for services (e.g., percent of people with disabilities who contributed to the cost of the service) or participation of users in the planning of rehabilitation services (percent of assessment and planning exercises involving community members – i.e., people with disabilities, see full indicator list).

In Nepal, the “community” was defined as the population living in areas served by rehabilitation centres rather than only existing or potential service users per se. Community capacity was perceived as the capacity of community organisations to organise themselves (e.g., percent of disabled people organisations that have action plans) and integrate disability and rehabilitation into their activities at decentralised levels (e.g., percent of districts with District Disability Rehabilitation Committees and Village Disability Rehabilitation Committee, see full indicator list).

In terms of the enabling environment, in both countries, workshop participants recognised the importance of securing political commitment at the national level to develop and implement disability-related policies (e.g., in Nepal, the existence of a national action plan on rehabilitation) and allocate public financial resources to the rehabilitation sector (e.g., in Somaliland, percent of price covered by Government).

### Physical rehabilitation stakeholder networks in Nepal and Somaliland at 2-year follow-up

**Figure 3:**
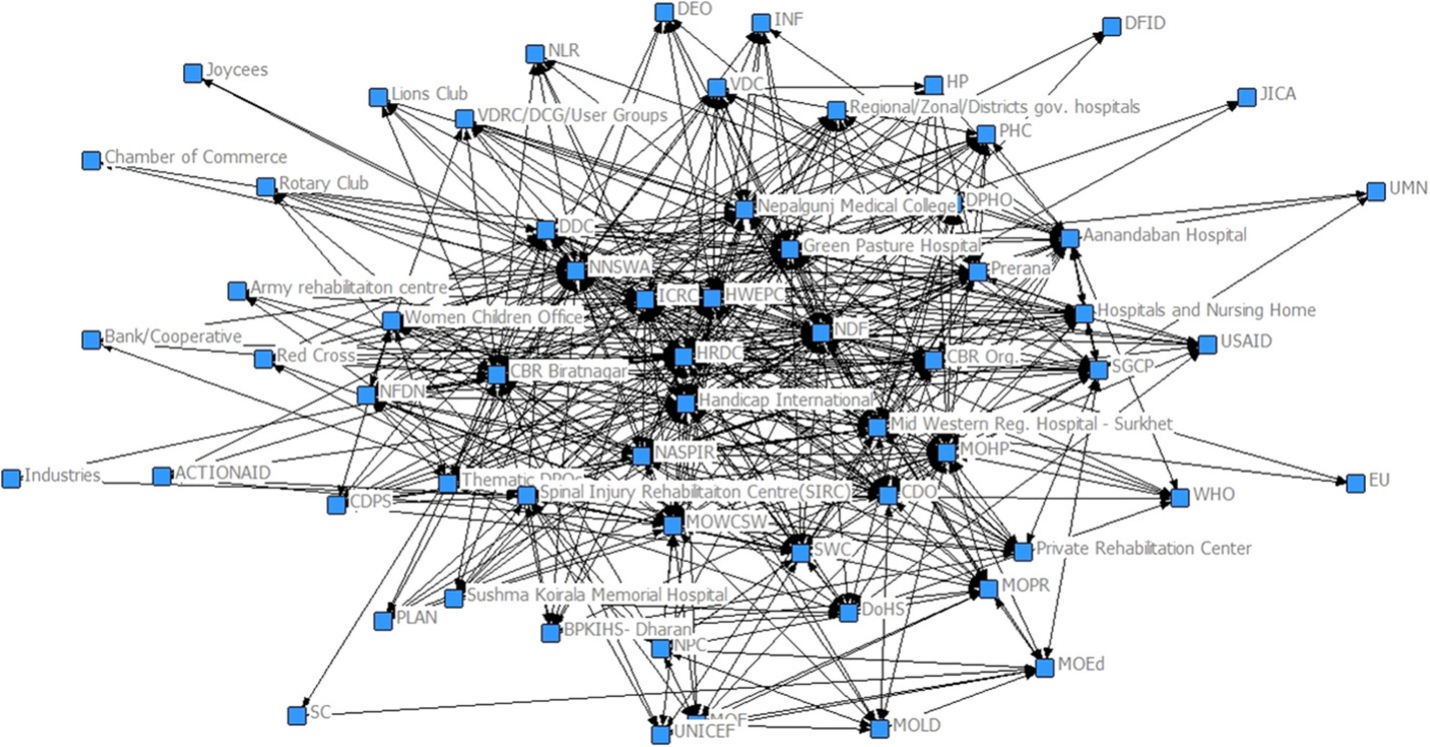
**The physical rehabilitation stakeholder network of Nepal in 2012 (follow-up).**

The system stakeholder network in Nepal became slightly less decentralised but denser over the 2 years from 2010 to 2012 with the appearance of seven new actors (including international and local organisations) (Figure 3).

**Figure 4:**
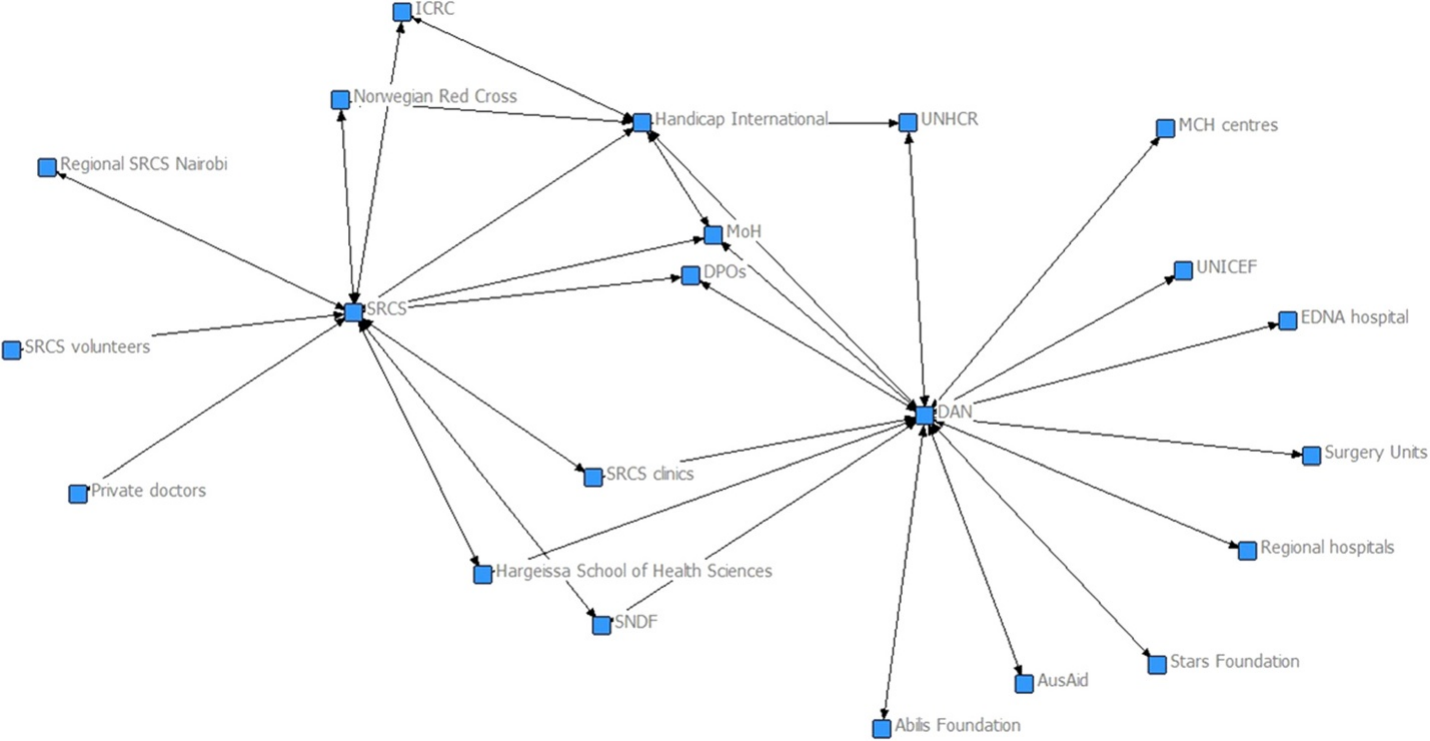
**The physical rehabilitation stakeholder network of Somaliland in 2012 (follow-up).**

In Somaliland, the major change was the disappearance of the relationship between the two principle brokers of the network (DAN and SRCS). Between the two SAP workshops, the two service providers never formally met to discuss the management of rehabilitation services or the governance of the system. The system became divided into two sub-systems with each service provider at the centre and surrounded by collaborative organisations. In spite of this system fracture, in 2012, the stakeholder network remained highly centralised (61%) and low in density (0.1) (Figure 4).

### Comparative 2-year sustainability progress and use of self-defined sustainability indicators

At 2-year follow-up there was little progress towards the local vision of sustainability and almost no use of the self-defined sustainability indicators in Somaliland. System stakeholders individually had not attempted to undertake organisational measurements or use this type of information in decision making, although most still perceived the indicators to reflect their vision of sustainability of the system. During this period, no organisation took the lead to coordinate follow-up of the SAP. Handicap International had sponsored the workshop and was perceived as the “owner” of the initiative by some actors, who expected the international NGO to continue the lead role. Handicap International, however, was reluctant to maintain active involvement in coordination since their overall objective was to hand-over their support to rehabilitation services to local partners. The Ministry of Public Health had limited capacity and political will to fulfil this role. The two main service providers continued to be focused on the survival and viability of their own individual organisations. Several system stakeholders characterised sustainability as “unachievable” under current conditions in Somaliland, without sufficient funding and support from the Ministry of Public Health. In Somaliland, by 2012, apart from a tax break on land for rehabilitation facilities, there was still no government funding allocated to provision of physical rehabilitation services.

In Nepal, all the sustainability indicators had been measured. A couple of months after the first workshop, Handicap International offered to take the lead and designate one of their project coordinators as the sustainability officer in charge of leading the sector on these issues. Through the initiative of this international organisation, an independent national consultant was commissioned in 2011 to collect further data to verify the estimates provided during the first workshop. During this assessment, 10 indicators could not be measured because of unclear definitions (i.e., no clear documentation on what the numerator and denominator were) and/or lack of existing data collection mechanisms. For example, data on the number of districts that have elaborated an action plan on disability is not routinely collected in information services primarily set-up to measure health service and human resource performance. Accessing this type of information would require comprehensively surveying district health offices and organisations, probably via field visits, which was beyond the financial resources available for the measurement exercise conducted in 2011. Data on the remaining indicators were published in a report circulated to the main actors of the rehabilitation sector (i.e., Ministry Officers, providers of rehabilitation services, and coordinators of rehabilitation programmes amongst international organisations).

Although it is difficult to attribute the precise causes involved, evidence from qualitative discussions with stakeholders suggested that the SAP, through this process of discussion, consensus-building, data collection, and information-sharing, appeared to support the advancement of system sustainability in Nepal by encouraging the participation of major stakeholders outside the rehabilitation sector in sustainability initiatives between 2010 and 2012. Following the first workshop and lobbying from the main actors of the network, three Nepalese Ministries (whose representatives had been invited to the final presentation of the sustainability indicators) agreed to invest funds to improve the functioning of the rehabilitation system as a whole instead of targeting specific rehabilitation centres. Their initial idea of creating new rehabilitation centres in the country was changed after the rehabilitation actors presented their vision of the sustainability of the sector. As a result, the Ministries agreed to support existing facilities and initiatives.

In contrast, in Somaliland, systems thinking and action was observed during this period only in an area peripheral to rehabilitation service delivery, on disability mainstreaming initiatives to increase government involvement in wider disability programming in the social sector [58]. This involved extensive collaborative work with actors across the rehabilitation system, under the auspices of the Ministry of Labour and Social Affairs. Partly, motivations for doing this work appeared to be in response to specific funded opportunities through government and international organisation channels. These, however, required a far lesser commitment of financial resources than opportunities for reform of rehabilitation services would have. There was also little evidence to suggest that information created during the SAP was used in these mainstreaming initiatives. While potentially laying the groundwork for systemic work on physical rehabilitation services in the long term, in the shorter term, over which this study was conducted, we found little evidence of systemic thinking helping to resolve the problems rehabilitation actors described facing to achieve their vision of sustainability in Somaliland.

## Discussion

In this study, we compared the selection and use of self-defined sustainability indicators in two countries to analyse the influence of contextual factors and social network structure on the development of physical rehabilitation systems. Our assumption was that baseline differences between the two networks as well as the nature of relationships between actors would influence the way the sustainability indicators would be defined and used during follow-up.

The definition of key sustainability indicators was implicitly influenced by network characteristics and actors’ perception of their own system. In Nepal, the stakeholder network at baseline was decentralized and dense with a wide diversity of stakeholder types. Here, vision of the future system was inherently systemic, including concerns about the coverage of services but also how the actors of the system work together and how integration of new actors could increase system impact. The actors of the rehabilitation sector who were interviewed recognised the importance of creating unity between all the actors of the network in order to more effectively negotiate with national authorities and donors. Hence, a national body representative of all the rehabilitation providers (NASPIR) was created in Nepal. In Somaliland, where the stakeholder network was centralized in the capital city and low in density (few stakeholders and brokers), the vision of the future physical rehabilitation system was constructed around the two rehabilitation facilities, positioned at the very centre of the system, and mainly represented their perspective. They focused on the extension of the services towards the provinces.

Social network analysis provides tools to identify knowledge brokers, i.e., individuals who create links between different groups within a system, such as between users and researchers, which was the case of the two rehabilitation centres in Somaliland [45]. The brokers in a health system also help coordinate actors in times of crises or shocks [59]. Other actors essential to the diffusion of innovations, such as opinion leaders, champions, or change agents, can variously be identified through the number of links they have with their peers or non-peer actors at different levels of the health system [60, 61]. One assumption from social network analysis is that the position of actors in a network determines their capacity to access and diffuse knowledge and information or, in other words, control the flow of information [62, 63]. A network with a central structure, such as Somaliland’s, has more capacity to coordinate actors and provide a rapid response, which may be very important during humanitarian crises [64]. However, in Somaliland, during the 2-year post-conflict period that we observed, the central position of the brokers in this much centralised network blocked the circulation of information and the use of sustainability data. Promising developments within the wider disability social movement in the country may, however, help overcome some of these circulation blocks between brokers in the future if more actors are brought into the network, thereby de-centralising information and decision making in the system.

The use of self-defined sustainability indicators by the system was also influenced by individual actors’ survival strategies. At 2-year follow-up, the dense network in Nepal became even stronger as actors prioritised integration of services for organisational growth and survival and was an enabling factor in the utilisation of sustainability indicators [58]. The emergence of a local champion, the sustainability coordinator, who was granted legitimacy by professional organisations and financial support by an international organisation, facilitated communication necessary to continue system sustainability work in Nepal within the rehabilitation sector. On the other hand, in Somaliland, the changing nature of relationships between the two main brokers of the networks completely disrupted the circulation of information between actors due to the highly centralized, low-density structure of the rehabilitation sector. This resulted in no follow-up activities to monitor or use the self-defined indicators. The space and time horizons [11, 32], which Somaliland stakeholders used to think about sustainability, shrank dramatically between 2010 and 2012 due to the interruption of international funding in the country. In formal network analysis terms, the “relationship” between the two main actors of the system disappeared in 2012 after they realised that their main and pressing priority was the survival of their own organisations. Midgley [65] showed that the decisions of individuals are primarily influenced by their survival instinct. Even well-documented evidence-based data cannot influence the decisions of an individual if the decision in conformity with the evidence represents a threat to his/her own interests and survival (e.g., professional career, family situation, or life threatening situation) [66]. When brokers shrank their sphere of intervention from the system to an organisation, the centralised stakeholder network in Somaliland, by nature of the relationships between brokers, suffered a bottleneck and therefore a barrier to systems thinking.

As this study demonstrates, adopting a system thinking approach involves at least three elements. First, it is essential to understand the choices and decisions being made by individual actors; second, to understand the positions of actors within the system, recognising that the choices of some actors have disproportionate influence on the system as a whole; third, it is important to understand the wider context affecting changes in the system over time (i.e., the existing social networks and the relationships between actors), recognising that systems are dynamic, social entities which are in constant mutation or adaptation [67].

## Conclusions

The highly centralised structure of the social network in Somaliland had potential to help rapidly diffuse information between actors, which might be very useful in contexts of emergency (conflict or natural disaster). However, the rupture of relationship between the two central actors of the network completely disrupted the functioning of the rehabilitation sector and lead to the non-use of sustainability data in a sector that became the sum of dispersed actors. In Nepal, the cohesion between actors was maintained thanks to the role of a local champion and the injection of additional funds in the sector. The network remained very dense and decentralised and actors there appear to be gradually building a systemic vision of their sector, which takes account of data such as sustainability indicators for planning and negotiation purposes. These findings suggest that using sustainability indicators for a health system requires cohesion within the system between all (or most) actors, as well as an understanding, by actors, of the benefit of a collective vision for the sector. Contextual factors, such as the availability of funding for activities that primarily benefit the system rather than individual actors or organisations, can also support this. Further research is needed to analyse the different strategies that are required for health system interventions to alter the characteristics of social networks in social contexts for a collective good.

## Abbreviations

DAN: Disability Action Network
SAP: Sustainability Analysis Process
SRCS: Somaliland Red Crescent Society.
